# Position as Well as Velocity Dependence of Spasticity—Four-Dimensional Characterizations of Catch Angle

**DOI:** 10.3389/fneur.2018.00863

**Published:** 2018-10-26

**Authors:** Yi-Ning Wu, Hyung-Soon Park, Jia-Jin Chen, Yupeng Ren, Elliot J. Roth, Li-Qun Zhang

**Affiliations:** ^1^Department of Physical Therapy and Kinesiology, University of Massachusetts Lowell, Lowell, MA, United States; ^2^Department of Mechanical Engineering, Korean Advanced Institute of Science and Technology, Daejeon, South Korea; ^3^Department of Biomedical Engineering, National Cheng Kung University, Tainan, Taiwan; ^4^Rehabtek LLC, Glenview, IL, United States; ^5^Department of Physical Medicine and Rehabilitation, Northwestern University, Chicago, IL, United States; ^6^Department of Physical Therapy and Rehabilitation Science, Department of Orthopaedics, University of Maryland, Baltimore, MD, United States; ^7^Department of Bioengineering, University of Maryland, College Park, MD, United States

**Keywords:** quantitative evaluation, spasticity, muscle, stroke, catch angle

## Abstract

We investigated the muscle alterations related to spasticity in stroke quantitatively using a portable manual spasticity evaluator.

**Methods:** Quantitative neuro-mechanical evaluations under controlled passive elbow stretches in stroke survivors and healthy controls were performed in a research laboratory of a rehabilitation hospital. Twelve stroke survivors and nine healthy controls participated in the study. Spasticity and catch angle were evaluated at 90°/s and 270°/s with the velocities controlled through real-time audiovisual feedback. The elbow range of motion (ROM), stiffness, and energy loss were determined at a slow velocity of 30°/s. Four-dimensional measures including joint position, torque, velocity and torque change rate were analyzed jointly to determine the catch angle.

**Results:** The catch angle was dependent on the stretch velocity and occurred significantly later with increasing velocity (*p* < 0.001), indicating position dependence of spasticity. The higher resistance felt by the examiner at the higher velocity was also due to more extreme joint position (joint angle) since the spastic joint was moved significantly further to a stiffer elbow position with the higher velocity. Stroke survivors showed smaller ROM (*p* < 0.001), higher stiffness (*p* < 0.001), and larger energy loss (*p* = 0.005). Compared to the controls, stroke survivors showed increased reflex excitability with higher reflex-mediated torque (*p* < 0.001) and at higher velocities (*p* = 0.02).

**Conclusion:** Velocity dependence of spasticity is partially due to joint angle position dependence with the joint moved further (to a stiffer position where higher resistance was felt) at a higher velocity. The “4-dimensional characterization” including the joint angle, velocity, torque, and torque change rate provides a systematic tool to characterize catch angle and spasticity quantitatively.

## Introduction

Spasticity commonly occurs to patients with neurological disorders, such as stroke, spinal cord injuries, cerebral palsy, and multiple sclerosis (1–3). Spasticity is commonly defined as “a motor disorder characterized by a velocity-dependent increase in tonic stretch reflexes (muscle tone) with exaggerated tendon jerks, resulting from hyperexcitability of the stretch reflex, as one component of the upper motor neuron syndrome” (4). Various measures have been used to assess muscle alterations associated with spasticity. In the clinical setting, spastic muscle is usually evaluated by grading the resistance to a passive stretch felt by a clinician using the Ashworth scale, modified Ashworth scale (MAS), and the Tardieu scale (5, 6). The felt resistance could be caused by a combination of neural and peripheral origins (i.e., biomechanical factors such as soft tissues or muscle properties). Although clinical measures are convenient to use, they can be subjective, less sensitive and qualitative rather than quantitative to varying degrees. Previous studies raised questions about reliability of the MAS assessment of spasticity (7–10). On the other hand, the Tardieu scale has been suggested as an alternative to the MAS (6). Tardieu scale is conducted using various stretch velocities rather than using only one velocity in MAS while determining the angle where resistance felt (i.e., catch angle). It is argued that the MAS does not differentiate between spasticity and contracture, while the Tardieu scale is not confounded by the presence of contracture (11). However, with either scale, determinations of the catch angle (12, 13) and range of motion (ROM) are influenced by stretch velocities and stretch force and subject to errors in reading the joint angle during assessments.

A quantitative assessment with controlled passive stretches is needed to improve the reliability of the clinical measures. Well-controlled quantitative measures, based on motorized mechanical perturbations and electrophysiological approaches, are mostly used in laboratory settings (5, 14–16), but size and ease-of-use issues limit their applications in clinical settings (17–19). Several portable devices have also been developed and spasticity evaluations were performed by deriving viscous neuro-mechanical properties of the limb from passive movement kinematics and joint reaction torques (19–22). However, those measurements did not translate easily to the common clinical assessments of ROM and catch angle. Reflex threshold measured in joint angle during passive movement has been used effectively to evaluate spasticity by investigating the onset of muscle activation to applied disturbance (23–25).

In spite of the current development of spasticity quantification, in clinical setting thus far, clinicians evaluate spasticity based on how much resistance they feel as well as where they feel the reactive resistance while manipulating the joint quickly. The relation with regard to velocity dependence between stretch-induced muscle activation onset and the resistance (catch) felt by the clinicians in the stroke survivors has not been investigated thoroughly. Furthermore, it is not clear whether catch angle is also joint angle position dependent. In other words, whether joint angle might play a role in the increasing resistance felt by clinicians at a higher velocity and being judged as velocity-dependent spasticity is uncertain. A comprehensive but simple way considering stretch velocities, reflex-mediated muscle torque and joint angle is needed to assist clinicians understand the muscle alteration due to neurological disorders and interventions. Therefore, the purpose of this study was to introduce an innovative and quantitative way to depict the spasticity according to the concept of Tardieu scale and further examined the contribution of joint angle position dependence to the catch felt by the examiner.

## Materials and methods

### Subjects

Twelve chronic stroke survivors (53.0 ± 8.5 years old, ten males and two females) who had a stroke more than 1 year (9.3 ± 5.6 years) and nine healthy controls (51.4 ± 24.9 years old, nine males) were included in this study. The stroke survivors with elbow flexors spasticity were recruited in the study. The subjects who had shoulder or elbow contractures were excluded from the participation. Table 1 depicts the characteristics of the stroke survivors. The healthy controls had no history of neurophysiologic or musculoskeletal disorders. The study was approved by the Institutional Review Board of Northwestern University. All subjects gave written informed consent before the experiment.

**Table 1:** Characteristics of subjects.

**Subject No**.	**Gender**	**Age (years)**	**Years since the onset**	**Hemiparetic side**	**MAS**
Sbj1	M	60	10	R	2
Sbj2	M	38	9	R	2
Sbj3	M	62	6	R	1
Sbj4	M	38	7	R	1
Sbj5	M	57	9	R	1
Sbj6	M	58	26	L	3
Sbj7	M	53	9	R	3
Sbj8	M	50	6	R	3
Sbj9	M	70	2	L	1
Sbj10	F	54	9	R	3
Sbj11	F	48	12	R	2
Sbj12	M	48	6	R	3

### Instrumentation

**Figure 1:**
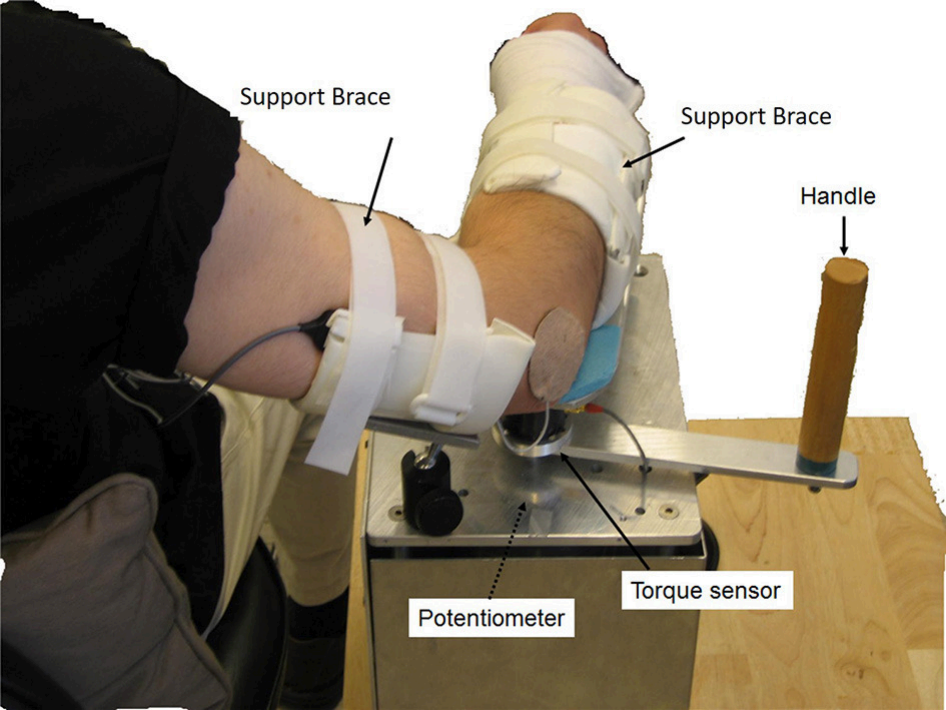
Experimental setup. The participant sat next to the MSE with 75° shoulder abduction. The participant's upper arm and forearm were then strapped onto the supporting braces with the elbow flexion axis aligned with the MSE rotation axis. A torque sensor and a hollow-shaft potentiometer were used to measure the joint torque and joint position, respectively. Surface EMG electrodes were used to record EMG signals of biceps brachii and triceps brachii.

The manual spasticity evaluator (MSE) used in this study was set up as a portable device to assess spastic muscles. A torque sensor (Transducer Techniques, CA, USA) and a hollow-shaft potentiometer (Vert-X51, Contelec AG, Switzerland) comprising MSE, were used to measure joint torques and joint positions respectively (26, 27). Adjustable braces and supports were used to position the forearm and upper arm properly with respect to the MSE (Figure 1). Two mechanical stops were used to restrict the moving range of device that prevents over-stretching of the spastic joint. Biceps and triceps muscles activations were monitored using surface EMG electrodes (Bagnoli-8, Delsys Inc., Boston, USA). The torque, position and EMG signals were sampled at 1,000 Hz with a 16-bit resolution. A custom data acquisition program was used to provide real-time audio and visual feedback to help an examiner control the peak stretch velocity and the peak stretch torque (terminal torque) (26). When the examiner passively moved the subject's forearm, the target velocity and torque profile with boundary lines (e.g., ±10% of target velocity or target torque), as well as the instantaneous joint velocity and torque were displayed on the monitor. At fast velocities (90°/sec and 270°/sec), the data acquisition program provided audio feedback for controlling peak velocities during passive stretching. At the slow velocity of 30°/sec, audio feedback was used to indicate that the designated torque limit had been reached and to stop the passive stretching. By doing so, the applied stretch force could be consistent while quantifying the spastic muscle each time.

A test-retest reliability investigation of the MSE was performed on the healthy controls by the same rater twice with 1 day apart. Excellent reproducibility was found in measures derived from fast stretching (ICC = 0.88) and from slow stretch (ICC = 0.89 for passive ROM, 0.84 for stiffness and 0.75 for energy loss). Excellent intra-rater reliability was also shown while using MSE in the pediatric population (28, 29).

### Experimental procedures

In a quiet room, a therapist assessed the spastic elbows of the stroke survivors using MAS (30). The therapist followed the procedure described previously (31) with the exception of the body position. In the current study, the subjects sat upright comfortably instead of lying down. The therapist stabilized the upper arm by holding it proximal to the elbow and moved the forearm in a quick passive motion (~1 sec) throughout the available elbow range of motion from the end of flexion to maximal extension.

During the experiment, the subject sat next to the MSE with the elbow flexion axis aligned with the rotation axis of the MSE. The shoulder was positioned at 75° of abduction and the forearm and upper arm were secured to the supporting braces. Surface EMG electrodes were placed on the biceps brachii and triceps brachii with the reference electrode placed on the lateral epicondyle.

In the clinical practice, the modified Tardieu R1 is the angle of the catch thought to be due to induced stretch reflex at an as fast as possible velocity. The passive ROM (R2) is graded under a slow velocity, which would not trigger the stretch reflex (12). In the current study, we chose 30°/sec as the slow velocity to measure R2 and chose two fast velocities (90 and 270°/sec) to detect the catch angle (R1) using MSE. The slow velocity of 30°/sec was chosen because it did not induce stretch reflex during manual tests that may confound the R2 measurement. Two high velocities (90 and 270°/sec) were chosen because 90° (right angle) and its multiples are relatively easier for the rater to perceive during manual tests.

Initially, the torque and position offset were recorded with the subject's elbow in the neutral position, defined as the position where subjects felt the most comfortable, not being stretched or restrained (79.6° ± 10.9° elbow flexion for stroke survivors, and 75.0° ± 6.5° elbow flexion for healthy controls with full extension defined as 0° elbow flexion). To determine the passive ROM and stiffness of the elbow, we then moved the elbow at 30°/sec until reaching a pre-defined torque (3 Nm) or a mechanical stop. Three trials of passive stretch separated by 1 min were performed at each of 90°/s and 270°/sec to evaluate the velocity-dependence of spasticity and catch angle. With practice, the examiner was able to control the peak velocity of passive stretch to match the target velocity as shown on the display (±10% of target velocity). One stretch cycle was defined from full flexion to full extension then back to full flexion. Since spasticity may be altered by repeated stretching (32), the elbow was not stretched more than three cycles in each trial. We also instructed the subjects relax during the tests. If voluntary muscle contractions to assist the stretch were detected by the examiner and shown as intermittent or continuous EMG activities of triceps brachii muscles, and/or EMG activities of the biceps brachii preceding the stretch initiation, the trial was discarded. The examiner would then let the subject rest before stretching the joint again.

The MAS scores, the biomechanical measures and MSE-measured R1 and R2 were taken by the same examiner.

### Data analysis and statistics

Biomechanical Measurement: Torque and position signals were filtered with a low-pass cutoff frequency of 50 Hz. The derivative of torque with respect to time, dτ(t)/dt was calculated from the acquired torque signals. All the biomechanical measures captured at 30°/sec were determined within the torque limits of 3 Nm, including the passive ROM [also described as the R2 angle (12)], elastic stiffness (K), energy loss, and elbow flexor torque at several joint angles. Stiffness, K, was defined as the slope of the torque-angle relationship of ascending arm at 70° of elbow flexion, a common range among the subjects. The energy loss was the area between the ascending and descending limbs of the torque–angle curve during rotation (5). Since different subjects had different ROMs and limb inertias, the energy loss was normalized by the transverse inertia and the individual's range of motion. The transverse inertia was estimated from the length of the forearm, the perimeter of the elbow, and maximum forearm and wrist circumference (33, 34). Joint angle position dependence of the resistance torque was evaluated at three selected elbow flexion angles (45°, 60°, and 75°) in the two groups and normalized by the body weight and height (35). The angles were near the end of stretch and around the common range (70°).

EMG Signal Processing: The EMG signals were filtered with a passband of 10–450 Hz. To determine the onset of muscle activations (reflex-mediated responses) for verifying the catch angle, EMG signals were presented in a linear envelope (LE) form: with the filtered EMG data full-wave rectified and then low-pass filtered at 10 Hz. The onset of the reflex EMG was determined when the amplitude of the EMG LE was larger than the mean plus three standard deviations (SD) of the background EMG (36). The background EMG was measured during the quiescent period before the passive stretch. The elbow flexion angle corresponding to the reflex EMG onset was thus determined.

**Figure 2:**
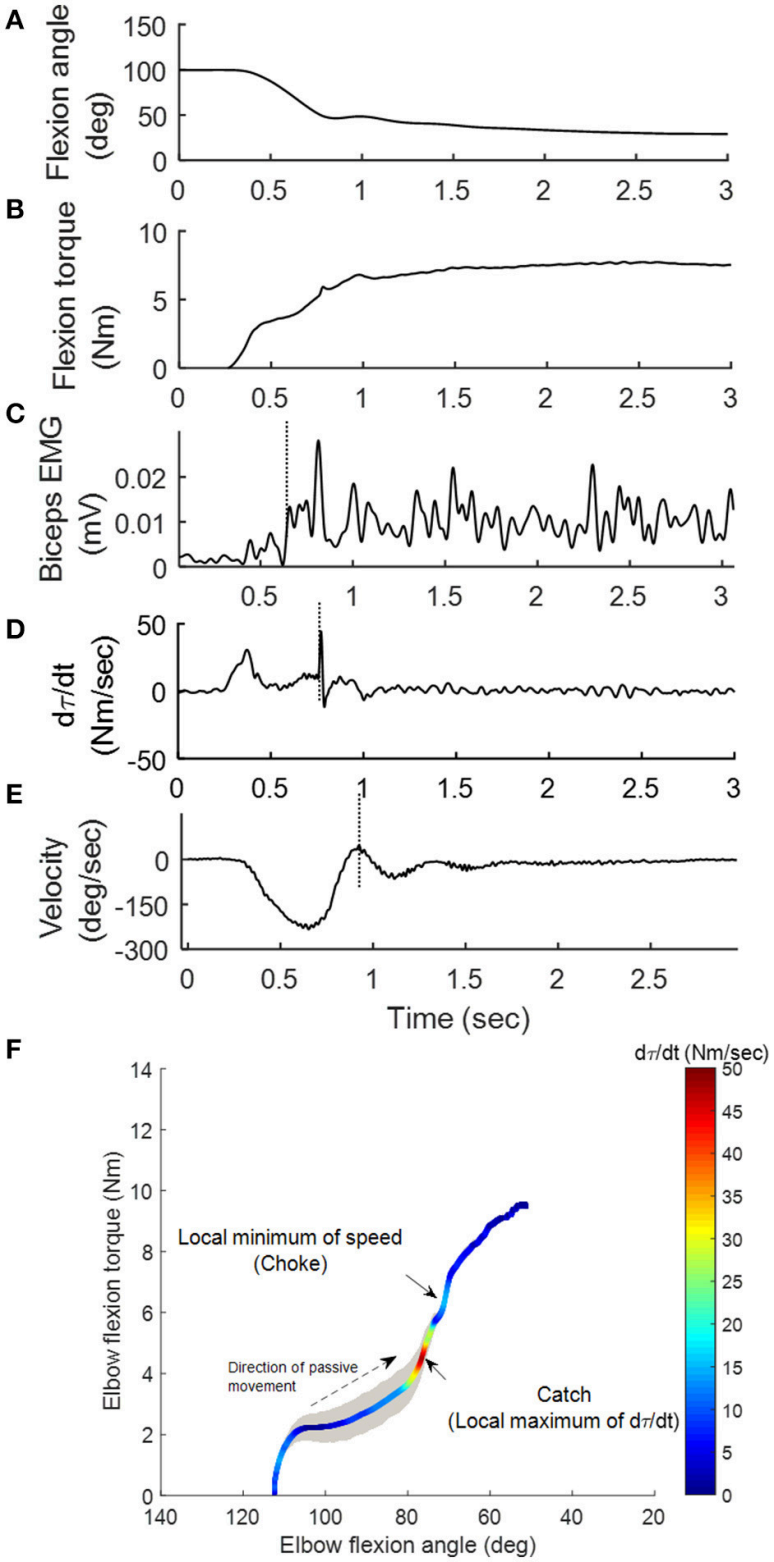
Representative kinematic, kinetic and EMG signals during the passive stretch. As the elbow was moved from 100° flexion to extension **(A)**, the elbow flexion torque τ **(B)** generated by the examiner on the stretched flexor muscles increased accordingly. Sequentially, the stretch induced a reflex response in the biceps (vertical line in **C**). The operator felt the sudden increase in resistance (vertical line in **D**) during the “catch,” and responded by decreasing the stretch velocity (vertical line in **E**). A four-dimensional display (**F**) was developed to illustrate the aforementioned events.

Catch Angle Determination and Characterization of spastic muscles: Catch angle is where a sudden occurrence of increased muscle activations in response to a quick passive stretch, which leads to an abrupt stop or increased resistance (torque) before the joint rotation reaches the end of ROM (37). This behavior can be captured using MSE as shown in Figure 2: the elbow flexor torque increased with elbow flexors being stretched (flexion angle decreased in Figures 2A,B). When the passive stretch triggered a stretch reflex (EMG firing shown in Figure 2C), the elbow flexors contracted strongly causing the abruptly increased torque rate (*d*τ*(t)/dt*) as indicated by the second positive peak in Figure 2D where the catch angle was determined. Figure 2E shows that the examiner responded to the abruptly increased resistance with a decreased velocity to avoid over-stretching the joint (value of velocity changed toward zero). We then developed a systematic way to determine R1. Since *d*τ*(t)/dt* was affected considerably by the inertia during the initial acceleration (the first peak of *d*τ*(t)/dt*), the local minimum of velocity was selected as the first landmark (the dashed vertical line in Figure 2E), which occurred shortly after the catch. Next, the joint angle corresponding to the peak *d*τ*(t)/dt* in the 300-ms window preceding this landmark was determined as the catch, R1 (26). The ratio R1/R2 was derived through dividing the angular displacement between R1 and flexion angle by the overall ROM, to represent the portion, which is free from the catch. A four-dimensional display, including the variables of joint angle, velocity, torque (τ), and *d*τ*(t)/dt*, was developed to illustrate the events involved in the catch that provides a more comprehensive quantification of spastic muscles group around the tested joint (Figure 2F) at various stretch velocities. The width of the shaded area in Figure 2F represents velocity. Note the increased width as the high velocity was maintained. The torque increased as the elbow was moved into extension indicated by the dashed arrow. When a catch occurred, *d*τ*(t)/dt* increased abruptly and reached a local maximum (the relatively hot color of the *d*τ*(t)/dt* line). The abruptly increased resistance and the examiner's reaction to the catch resulted in a quick velocity reduction to a local minimum (choke, the narrow shaded area).

Statistics: Since the data was not normally distributed, non-parametric statistics (Friedman test) were used for comparisons of catch angles at different stretch velocities with a significance level of *p* < 0.05. To investigate differences in the biomechanical measures (ROM, stiffness, torque at three joint positions, energy loss) and the velocity-dependent properties of muscle compared between the control and CVA groups, a Mann-Whitney U-test with a significance level of *p* < 0.05 was conducted. Spearman correlation was used for correlating the MAS with all biomechanical measures as well as the catch angle. Correlation was significant at *p* < 0.05. The intra-class correlation coefficient was chosen as the test statistic to evaluate the test-retest reliability. The two-way mixed model of intra-class correlation coefficient was used. An intra-class correlation coefficient ≥ 0.75 indicated significant reproducibility (38). SPSS software (SPSS Inc., Chicago, Illinois) was used to perform all statistical analysis.

## Results

### 4-D characterization of catch angle

**Figure 3:**
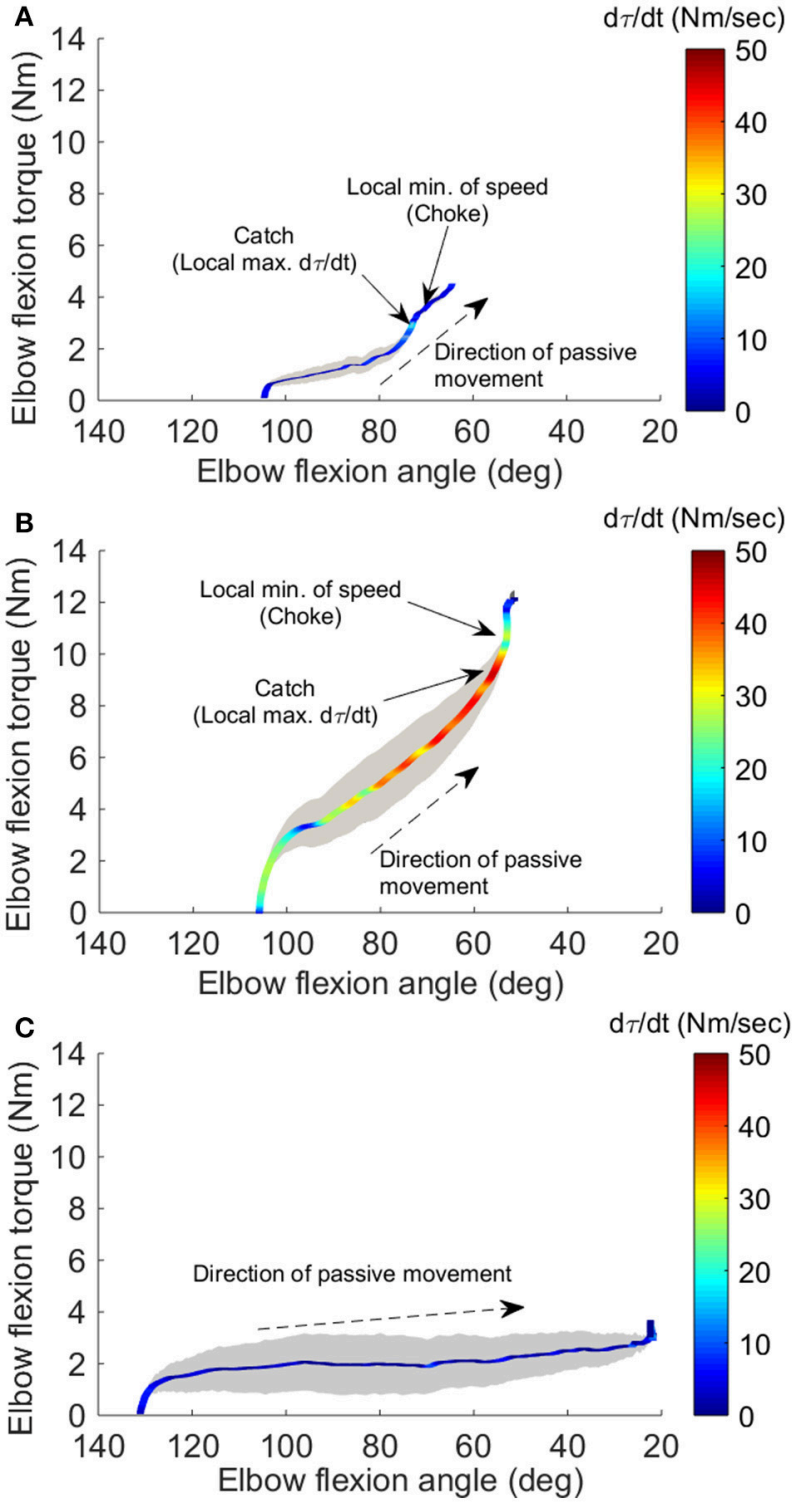
Four-dimensional characterization of the spastic muscles. Four variables: elbow flexion angle, elbow flexion torque, velocity, and torque change rate dτ(t)/dt, during the passive stretch were plotted simultaneously for a representative stroke survivor at 90°/sec **(A)**, at 270°/sec **(B)**, and a healthy control at 270°/sec **(C)**. The X-axis and Y-axis correspond to the elbow flexion angle and torque, respectively. The resistance torque generated by the stretched flexor muscles is positive. The dashed arrow indicates the direction of movement. Velocity is proportional to the width of the gray shaded area. The color of the line gives dτ(t)/dt, with the hot color (red) corresponding to a high rate of dτ(t)/dt. The two small arrows in (A) indicate the local maximum of dτ(t)/dt where the catch occurred and local minimum of velocity resulting from the examiner's reaction to the abruptly increased torque, respectively.

Figures 3A,B show the representative result of spasticity and catch angle evaluations at velocity of 90°/s and 270°/s respectively. Figure 3C shows the representative curve in a healthy control, the dτ(t)/dt remained at a constant lower value (below 10 Nm/s) throughout the available range of motion. In addition, for healthy controls the joint resistance was much lower when compared to stroke survivors (*p* < 0.05).

### Dependence of R1 on stretch velocity and velocity-dependent properties of spastic muscles

**Figure 4:**
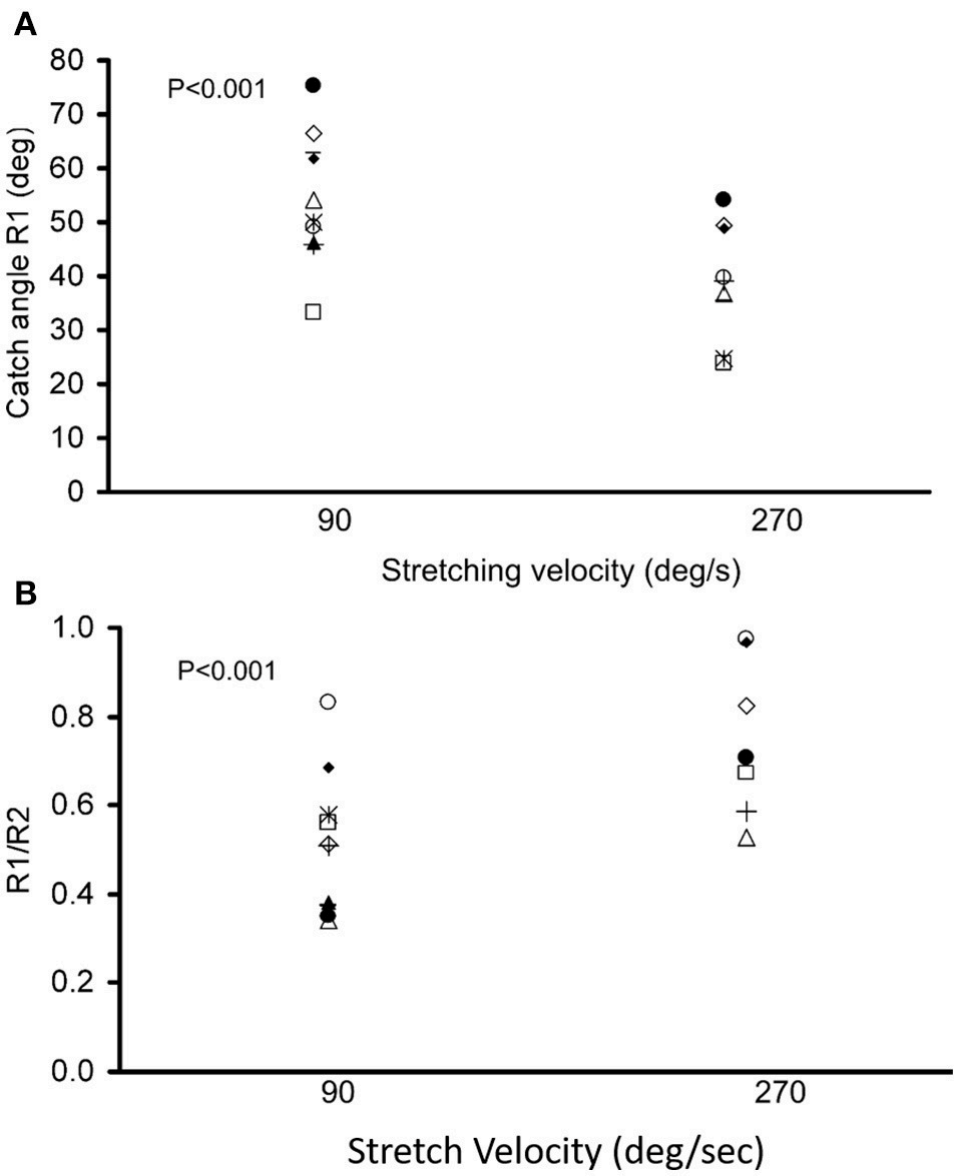
Dependence of catch angle on the passive movement velocity. The catch angle (R1) and the ratio of the angular displacement between catch angle and initial flexion angle over ROM (R1/R2) with a controlled torque limit are shown in **(A,B)**, respectively. Each symbol represents a stroke survivor. Friedman test was used for multiple comparisons of catch angles at different stretch velocities with a significance level of *p* < 0.05.

During the passive elbow extension, the catch angle in stoke survivors was significantly smaller (elbow more extended, *p* < 0.001) with increasing stretch velocity (Figure 4A). This indicates that the catch angle occurred later at faster stretch velocities. Furthermore, the R1/R2 was significantly higher at higher stretch velocities (Figure 4B, *p* < 0.001). As expected, catch was not observed in any healthy controls. In the representative case shown in Figures 3A,B, catch occurred at 78.2° and 58.8° elbow flexion at the stretch velocities of 90°/s and 270°/s, respectively.

**Figure 5:**
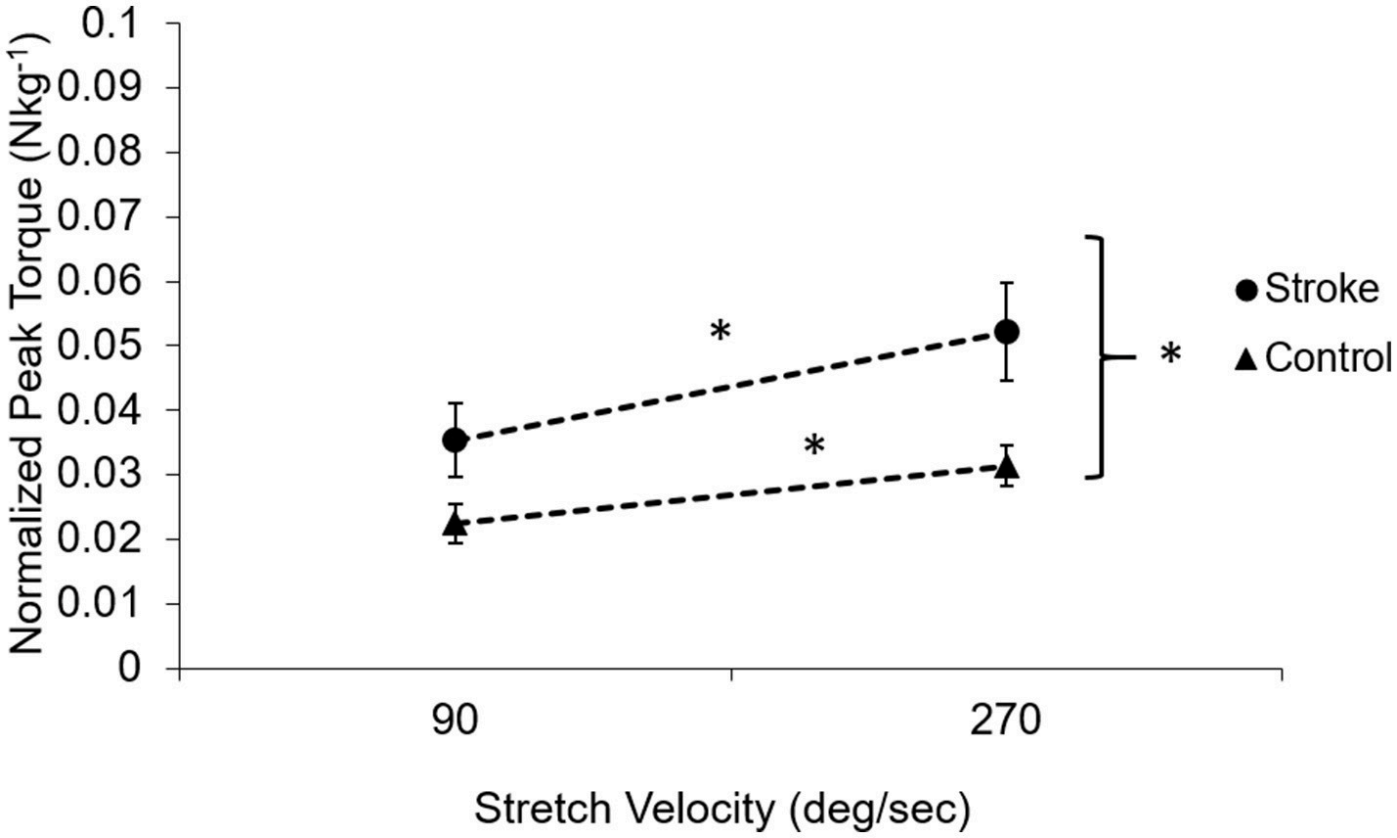
Velocity-dependent property of the muscle in subjects post-stroke (solid circles) and healthy controls (solid triangles). The dotted lines demonstrate the dependence on the velocity. Subjects post-stroke had a stronger velocity dependence compared to healthy controls (*p* = 0.02) and had higher torque at all velocities than healthy controls (*p* < 0.001). Asterisks (*) indicates significant differences between two velocities.

As a feature of spasticity, the peak resistance torque during a stretch increased with the increasing stretch velocity in the stroke survivors (*p* < 0.005, Figure 5). Healthy controls also showed increased peak resistance at the velocity of 270°/s when compared to the velocity of 90°/s (*p* = 0.005). The slope of the relationship between the peak torque and the stretch velocity was significantly higher in stroke (9.34 nu × 10^−5^ ± 4 × 10^−5^ Nkg^−1^deg^−1^s) than that in healthy controls (4.99 × 10^−5^ ± 2.95 × 10^−5^ Nkg^−1^deg^−1^s; *p* = 0.02).

### Biomechanical measures of the spastic muscles

**Figure 6:**
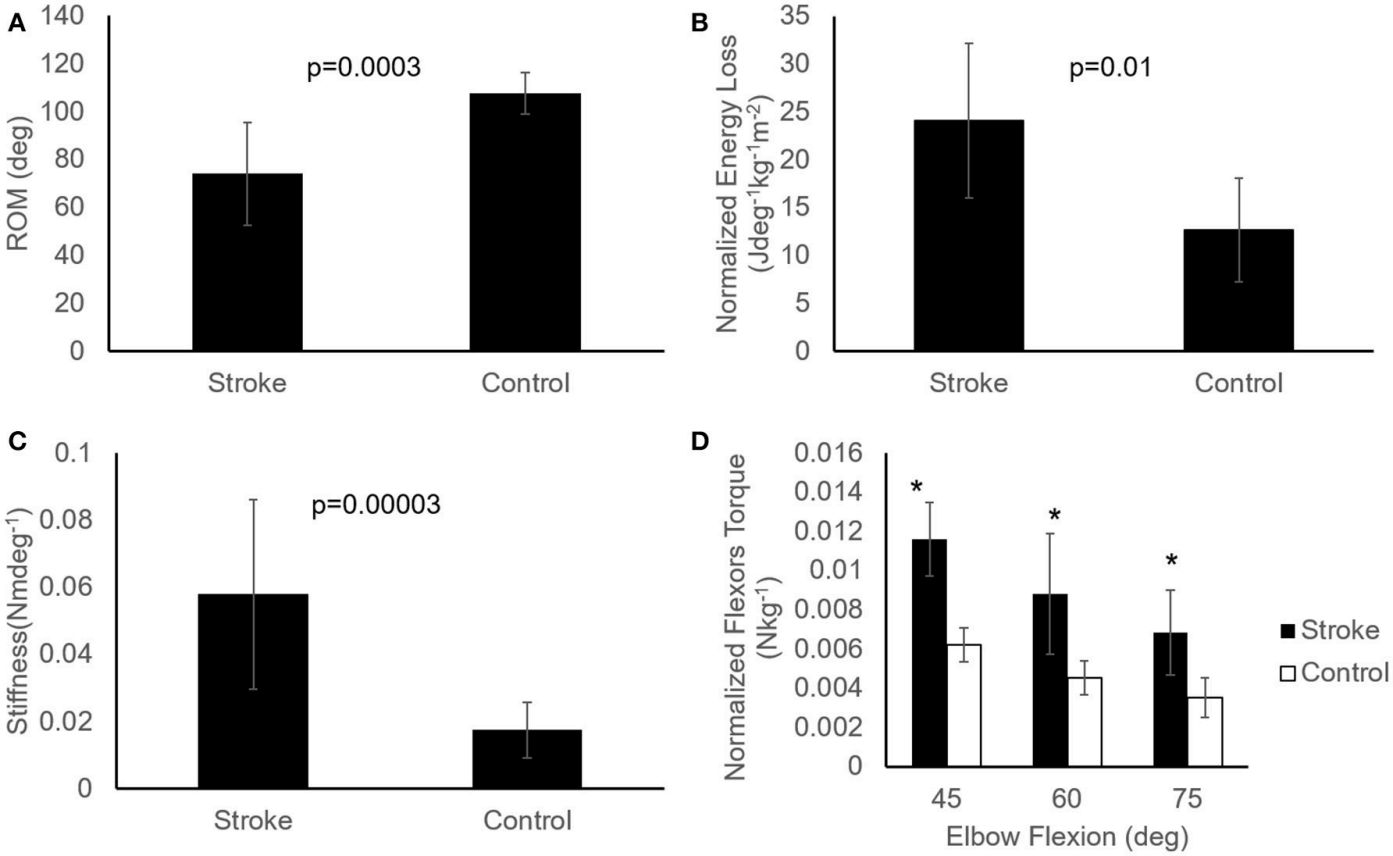
Comparisons of passive properties of elbow flexors between the healthy controls (*n* = 9) and stroke survivors (*n* = 12). Patients post stroke showed reduced ROM **(A)**, higher energy loss **(B)**, increased stiffness **(C)**, and increased resistance torque at several angles **(D)**, represented as mean ± SE (standard error of mean). Asterisks (*) indicate significant difference (*p* < 0.05) between the two groups **(D)**. Mann-Whitney U test with significance level of *P* < 0.05 was used to determine the differences.

**Figure 7:**
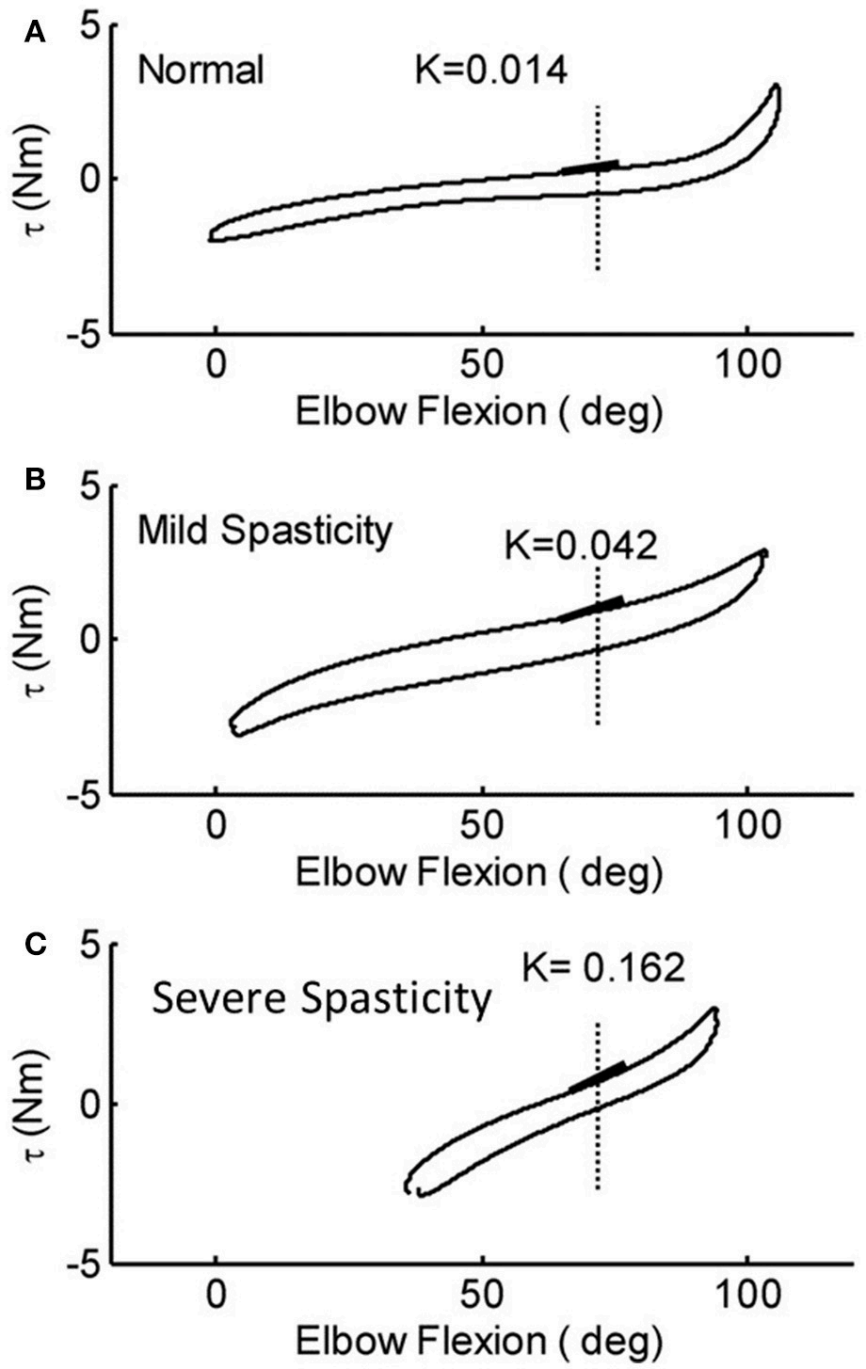
Representative torque-angle curves of stroke survivors [mild **(B)** and severe **(C)**] and a healthy control **(A)**. Full elbow extension = 0°. The slope of the thick black lines indicates the elastic stiffness of the elbow recorded at a common angle.

ROM measured at a controlled torque of 3 Nm was significantly reduced in the stroke survivors as compared to that of the healthy controls (74.2° ± 21.5° vs. 107.6° ± 8.7°, *p* < 0.001; Figure 6A). During extension with a 3 Nm torque limit, the stroke survivors stopped earlier at larger flexion angles (30.0° ± 17.6°) compared to healthy controls (10.2° ± 10.8°; *p* < 0.01). Figure 7 shows the examples of restricted ROMs for the severely spastic (MAS = 3), mildly spastic (MAS = 1) and healthy controls that were 36°-93°, 3°-104°, and −1°-106° elbow flexion, respectively.

Stiffness measured at a prescribed elbow flexion angle of 70° was significantly larger in stroke survivors when compared to healthy controls (Figure 6C; 0.058 ± 0.028 Nm/deg vs. 0.017 ± 0.008 Nm/deg, *p* < 0.001). The stiffness for the severely spastic, mildly spastic and healthy controls was 0.162 Nm/deg, 0.042 Nm/deg, and 0.014 Nm/deg respectively (Figure 7, slope of the lines).

Stroke survivors lost more energy (24.14 ± 8.07 Jdeg^−1^kg^−1^m^−2^) than healthy controls (12.66 ± 5.38 Jdeg^−1^kg^−1^m^−2^; *p* = 0.005; Figure 6B). In addition, stroke survivors showed higher torque compared to healthy controls at 45°, 60°, and 75° of elbow flexion (stroke vs. healthy: 0.0116 ± 0.001 Nkg^−1^ vs. 0.0062 ± 0.0008 Nkg^−1^, 0.0088 ± 0.0031 Nkg^−1^ vs. 0.0045 ± 0.00084 Nkg^−1^, and 0.0069 ± 0.0021 Nkg^−1^ vs. 0.0035 ± 0.001 Nkg^−1^, respectively; Figure 6D).

### Correlations between the MAS, R1 and biomechanical measures

Table 2 shows the correlations among the variables evaluated. The catch angle showed significant correlations with ROM (*r* = −0.685, *p* = 0.014) and with energy loss (*r* = −0.679, *p* = 0.047). The MAS showed significant correlations with ROM (*r* = −0.897, *p* = 0.001), as well as stiffness of the joint (*r* = 0.828, *p* = 0.006). Reflex-mediated EMG responses at different stretch velocities did not show any significant correlations with MAS, R1 or biomechanical measures (*p* > 0.05).

**Table 2:** Spearman correlation coefficient p (and *p*-value) between the parameters.

		**MAS**	**R1**	**EMG**	**ROM**	**EL**	**K**
MAS	ρ	1.000					
	*p*						
R1	ρ	0.539	1.000				
	*p*	0.054					
EMG	ρ	−0.223	0.405	1.000			
	*p*	0.282	0.160				
ROM	ρ	−0.897^**^	−0.685^*^	0.050	1.000		
	*p*	0.001	0.014	0.449			
EL	ρ	0.091	0.679^*^	0.086	−0.067	1.000	
	*p*	0.408	0.047	0.436	0.432		
K	ρ	0.828^**^	0.613	−0.319	−0.934^**^	−0.371	1.000
	*p*	0.006	0.072	0.269	0.001	0.234	

*MAS, modified Ashworth Scale; R1, catch angle; EMG, electromyography onset; EL, energy loss; K, stiffness*.

* indicates P < 0.05

** *indicates P < 0.01*.

## Discussion

This study demonstrates 4-D plot, a comprehensive, and systematic way, to investigate the group of spastic muscles around the elbow. Spasticity-related biomechanical characteristics, including joint ROM, joint torque, stiffness and energy loss, at various controlled velocities can also be acquired at the same single setting. During a controlled slow stretch, the MSE assesses biomechanical properties of the joint including the ROM, stiffness and energy loss using a controlled slow stretch and determine the catch angle at controlled fast stretch velocities.

Convenient spasticity quantification has been a challenge. In order to evaluate stretch reflex responses accurately, the way to elicit spasticity should be standardized. Many factors including the pre-activation of muscles, position of the joints involved, and applied stretch torque and velocity, as well as the experience of clinicians may result in different outcomes and interpretations. Clinical measures, such as the MAS or Tardieu scales, have been used to identify the catch angle during passive stretch. However, the angles determined using these scales may not be accurate; they generally occur later than a biomechanically-detected catch and suffer from poor inter-rater reliability (39). As shown in Figures 2, 3, the examiner reacted to the abruptly increased joint resistance by slowing or stopping the passive stretch. However, the catch occurred up to 300 ms prior to this slowing or stopping point. Therefore, peak *d*τ*(t)/dt*, instead of the stopping point, should be used as the indicator of the catch angle. In the current study, the instantaneous velocity change along with *d*τ*(t)/dt* were used to determine the catch angle reliably and to minimize potential human error. As one can see in Figures 2, 3, the human's reaction characterized as local minimum of speed (choke) was further away from the catch determined by *d*τ*(t)/dt*. The discrepancy between human's reaction and the true catch implies the potential human errors in the subjective clinical measures of “catch angle.” As seen in the examples in Figures 2, 3, the differences ranged from 3 to 8°. In a clinical setting, the catch angle reading is usually from eyeballing of a goniometer moving with the joint, which may introduce even a larger error in determining the catch angle. It should be noted that another peak of torque change rate, which occurred earlier during the passive stretch, was when an examiner overcame the resistance from the limb inertia and was not related to catch angle.

Velocity-dependent increase in muscle tone is a key attribute to spasticity as shown in the current study as well that the stretch velocity has obvious effects on the normalized peak torque of catch (larger slope in Figure 5). However, the velocity dependence of spasticity might be partially due to joint angle position dependence. The delayed catch angle associated with fast velocities in our study showed the joint angle position dependence of the increased resistance. At a faster stretch velocity, the joint was quickly stretched further into an angle position where higher resistance existed. Assuming reflex-mediated torque developed 60 ms after the stretch reflex was triggered, the joint would have been moved 10.8° further in 60 ms at 270°/s as compared to the stretch at 90°/s ((270°/s-90°/s) × 0.06 s = 10.8°). The extra 10.8° moved the joint to a stiffer position, which might make the examiner feel higher resistance at a faster velocity. Similar results were reported that greater catch angles were associated with higher applied angular velocities (28, 40). Velocity-dependence of the catch angle further confirms that a standardized method to evaluate spastic muscles is essential since the interpretation of catch angle can be confounded by the stretch velocities.

In the current study, passive properties were measured under real-time feedback control by moving the elbow slowly without eliciting a reflex response. The lack of a reflex response was corroborated by silence in the EMG signals of the stretched muscle. Significant changes in the passive properties of the spastic elbow of stroke survivors were observed when compared to healthy controls, including increased stiffness and flexors resistance, decreased ROM, and increased energy loss (Figure 6). Similar changes were also found in the spastic ankle of stroke survivors with hemiparesis (5). In general, the changes in stiffness and ROM were consistent with what have been reported previously (14, 41, 42). Since the supporting braces fixed to the MSE might hinder the ROM near the end of flexion, the value of elbow ROM shown in current study was smaller than the observations in previous studies (43–45). In addition, Figure 7 also shows that the reduced ROM was not only in one end, the stroke survivors may lose the range toward flexion or extension that can be relevant to daily functions.

The correlation between passive stiffness and MAS demonstrates that the MAS is more closely related to the passive stiffness of the joint than to joint spasticity, even though it has been commonly used for assessing spasticity in both clinical and laboratory settings. MAS only includes as single stretch velocity and is scored by the amplitude of joint resistance that potentially make MAS reflect passive stiffness over spasticity. Because the felt joint resistance could be from either spastic responses and/or passive stiffness (46, 47) and without various stretch velocities those could not be distinguished. The consequence of this ambiguous assessment may mislabel patients who have increased passive stiffness alone as having spasticity and being treated with inappropriate interventions. Damiano et al. indicated that evaluating patients at different velocities may help to distinguish passive stiffness from spasticity (46), which we adopted in our developed method for spasticity quantification. Administering the Tardieu scale using the MSE could provide proper spasticity characterization under various controlled velocities. The intra-rater reliability of clinical assessments can be improved using the MSE that contains accurate sensors and provides real-time audiovisual feedback instead of the examiner's subjective manipulation and scoring.

## Limitations of the study

The brace of the system we used might prevent the all range of motion toward the end of flexion due to the contact of muscle bulks and brace. When there was no compromise using this system to assess the elbow flexors spasticity, one should interpret the passive flexion ROM with cautions. The sample size of the current study is relatively small. A larger size of sample should be considered in a future study.

## Ethics statement

This study was carried out in accordance with the recommendations of Northwestern University Institutional Review Board with written informed consent from all subjects. All subjects gave written informed consent in accordance with the Declaration of Helsinki. The protocol was approved by the Northwestern University Institutional Review Board.

## Author contributions

Y-NW, L-QZ, H-SP, and YR designed and carried out the experiment. Y-NW and L-QZ performed the data analysis. Y-NW, H-SP, YR, and L-QZ wrote the manuscript with support from J-JC and ER.

### Conflict of interest statement

The authors declare that the research was conducted in the absence of any commercial or financial relationships that could be construed as a potential conflict of interest.
